# Use of Chalcogenide-Semiconductor-Sensitized Titania to Directly Charge a Vanadium Redox Battery

**DOI:** 10.3390/nano10061137

**Published:** 2020-06-09

**Authors:** Tatiana Santos Andrade, Anastasios Keramidas, Panagiotis Lianos

**Affiliations:** 1 Department of Chemical Engineering, University of Patras, 26500 Patras, Greece; tsandrade@live.com; 2 Department of Chemistry, University of Cyprus, Nicosia 1678, Cyprus; akeramid@ucy.ac.cy

**Keywords:** photocatalytic fuel cell, vanadium redox battery, unmediated charging, TiO_2_, CdS, CdSe, ZnS, VOSO_4_

## Abstract

Unmediated charging of a battery using solar radiation is a very attractive project of solar energy conversion and storage. In the present work, solar energy was converted into electricity using a photocatalytic fuel cell operating with a chalcogenide-semiconductor-sensitized nanoparticulate titania photoanode and an air-cathode functioning by oxygen reduction. This cell produced sufficient energy to directly charge a vanadium redox battery functioning with a VOSO_4_ electrolyte and carbon paper electrodes. The whole system is characterized by ease of construction and simplicity of conception; therefore, it satisfies conditions for practical applications.

## 1. Introduction

One of the major issues related with the exploitation of solar energy is the need for energy storage. It is, of course, always possible to convert solar energy into electricity using classical photovoltaics and then charge a battery. However, photovoltaic panels, power converters and existing batteries are generally considered a costly combination of devices. A simplified solar energy converter combined with an inexpensive reversible energy storage system, is a very attractive perspective, which will make solar energy storage economically viable. In this respect, the present work deals with the use of a photocatalytic fuel cell (photo fuel cell, PFC) as a solar energy converter and the unmediated charging of a vanadium redox battery (VRB).

PFC is a fuel cell which is based on the photocatalytic oxidation of a sacrificial agent at the photoanode and the reduction of dioxygen at the cathode electrode. PFC then is a photoelectrochemical cell which consists of two electrodes; one anode and one cathode, in contact with an electrolyte [1,2,3,4,5,6,7,8,9,10,11]. The (photo)anode carries a semiconductor photocatalyst. Photons absorbed by the photocatalyst generate electron–hole pairs. Holes are consumed by oxidation of the sacrificial agent, the ‘fuel’, while electrons are collected by and flow in an external circuit. Water may act as a sacrificial agent, thus water may be oxidized producing oxygen; however, it is more effective to oxidize an added substance, which may as well be a waste. Electrons arriving at the cathode electrode assist reduction reactions, the most common being atmospheric oxygen reduction [12]. One of the advantages of a PFC is the conversion of solar energy into electricity concurrently consuming waste, thus, offering a double environmental benefit [3,8,11], i.e., solar energy conversion and degradation of wastes.

The choice of a VRB as an energy storage device is dictated by the simplicity, the reversibility and the functionality of vanadium redox batteries [13,14,15,16]. The interest in vanadium batteries has been refined in recent years as demand for energy storage has increased. The vanadium battery has a longer life span (20 years) than lithium batteries (5 years) and stores at least four times more energy. VRB can be recharged more frequently than other rechargeable batteries without any damage. Most importantly, at the end of the VRB life, it can be reused and thus does not raise disposal problems. A VRB is built into a two-compartment cell, the two compartments separated by an ion transfer membrane, and operates by the following reaction schemes:

VO^2+^ + H_2_O ↔VO_2_^+^ + 2H^+^ + e^−^   E_0_ = 1.004V vs. SHE (1)

V^3+^ + e^−^ ↔ V^2+^   E_0_ = −0.255V vs. SHE (2)

which are one-electron reactions, therefore, they may be realized without the need of powerful electrocatalysts. Indeed, such devices can operate with simple inexpensive carbon electrodes. In addition, they are based on one single material, VO^2+^, which is immune of any cross-compartment contamination issues. During charging, VO^2+^ is oxidized to VO_2_^+^ at the positive electrode (V^IV^→V^V^) and reduced to V^2+^ at the negative electrode in two steps. The first step is described by Equation (3)

VO^2+^ + 2H^+^ + e^−^ → V^3+^ + H_2_O (3)

and the second by Equation (2). It suffices then to introduce the same material in the two compartments of the cell, for example VOSO_4_, and proceed with charging. The progress of the VRB charging can be easily monitored by color changes: blue to yellow for the oxidation of VO^2+^ to VO_2_^+^ and blue to green to violet for the reduction of VO^2+^ to V^3+^ to V^2+^. 

**Figure 1:**
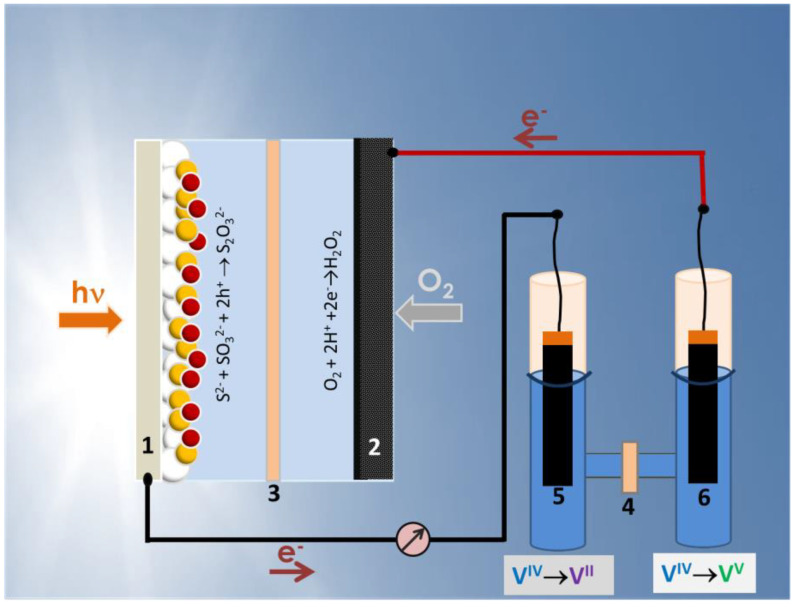
Schematic illustration of the photocatalytic fuel cell (left), the vanadium redox battery (right) and their connection: (1) photoanode; (2) air cathode; (3,4) ion transfer membranes; (5,6) carbon paper electrodes. Before charging, the battery cell is symmetric. After charging, the electrolyte associated with electrode (5) is reduced while the electrolyte associated with electrode (6) is oxidized.

Unmediated charging of the VRB means that the PFC is directly connected to the VRB without intermediates. In other words, the photoanode is connected to one of the electrodes of the VRB, which will cause reduction of the corresponding reagent. The cathode (air-electrode) of the PFC is connected to the second electrode of the VRB and the corresponding reagent will thus be oxidized (cf. Figure 1). The geometry of these connections may be important in practical applications but for the present case they may be simply effectuated by two short cables (cf. Figure 1). In order for the battery to be charged, first of all, the PFC must produce electricity without any external bias, preferably with open circuit voltage higher than the theoretical open circuit voltage of the battery, i.e., >1.259 V (cf. Equations (1) and (2)). It is then necessary to construct a PFC with such prerequisites and study its electric characteristics and then proceed to battery charging. These prerequisites were presently satisfied by employing a cell with a photoanode made of chalcogenide-semiconductor-sensitized nanoparticulate titania and a cathode electrode made of an air-breathing carbon cloth electrode.

## 2. Materials and Methods 

### 2.1. Materials

All reagents employed in the present work were purchased from Sigma-Aldrich, unless otherwise specified, and were used as received. Thus, transparent fluorine doped tin oxide electrodes (FTO, Resistance 8 ohms/square) were purchased from Pilkington North America (Toledo, OH, USA), carbon cloth from Fuel Cell Earth (Wobum, MA, USA), carbon black (from Cabot Corporation, Vulcan XC72, Billerica, MA, USA), carbon paper with hydrophobic layer from Quintech (H2315, Goeppingen, Germany) and Nafion membrane from Ion Power, Inc (N117, Newcastle, DE, USA).

### 2.2. Construction of the PFC

The PFC cell was a home-made device based on Plexiglas, which was divided into two compartments by a Nafion membrane, as schematically shown in Figure 1. The capacity of each compartment was 10 mL. It had two windows which were sealed by the two electrodes (photoanode and cathode). The anode compartment was filled with an aqueous electrolyte containing 0.25 M Na_2_S and 0.125 M Na_2_SO_3_, while the cathode compartment was filled with 0.5 M Na_2_SO_4_. The active area of each window was 3 cm^2^ (1.5 cm × 2 cm). Illumination of the photoanode was made with a Xe lamp, which provided approximately 100 mW cm^–2^ at the position of the catalyst. Light entered through a transparent FTO electrode.

### 2.3. Construction of the Photoanode

The photoanode electrode was made by depositing a 10 µm nanoparticulate titania film on an FTO electrode and it was sensitized by a combination of CdS, CdSe and ZnS quantum dots. The whole electrode was made by a standard procedure, which has been repeatedly published in the past, for example, in Reference [17]. In short, CdS quantum dots were formed in the mesostructure of titania by successive ionic layer adsorption and reaction (SILAR, 10 cycles), followed by CdSe formation by chemical bath deposition. Finally, a stabilizing ZnS layer was formed on the top by 2 additional SILAR cycles. Therefore, the photoanode was a ZnS/CdSe/CdS/TiO_2_/FTO electrode.

### 2.4. Construction of the Cathode Electrode for the PFC

The PFC functioned by oxygen reduction at the air-breathing (gas diffusion) cathode electrode, which was made of carbon cloth (CC) covered on the inner side by a hydrophobic layer of carbon black (CB). Deposition of the hydrophobic layer followed again a standard protocol [17].

### 2.5. Description of the Employed VRB

The vanadium redox battery used in the present work was an H-shaped reactor made of pyrex glass, also using Nafion as the ion transfer membrane (cf. Figure 1). The capacity of each compartment was 40 mL. Both compartments were filled with the same aqueous electrolyte containing 1 M VOSO_4_ and 2 M H_2_SO_4_. This is not a flow cell [13,14,15,16]. For practical reasons, no flow of electrolyte has been applied in the present case, which is sufficient for the present application. Two commercial carbon paper pieces carrying a layer of nanoparticulate carbon have been used as battery electrodes both during charging and discharging. Their active size was 1 cm × 2 cm. The reason these electrodes were different from the air-electrode used as the cathode in the construction of the PFC is because carbon paper is planar and geometrically stable, therefore, it can be immersed in an aqueous electrolyte. The catholyte, i.e., the electrolyte at the positive electrode, was deaerated by N_2_ flow before charging to avoid any possibility of unwanted reduction by oxygen.

### 2.6. Measurements

Electric measurements have been performed with the help of an Autolab potentiostat PGSTAT128N and reflection–absorption spectra with a Shimadzu UV-2600 absorption spectrophotometer equipped with an integration sphere.

## 3. Results and Discussion

**Figure 2:**
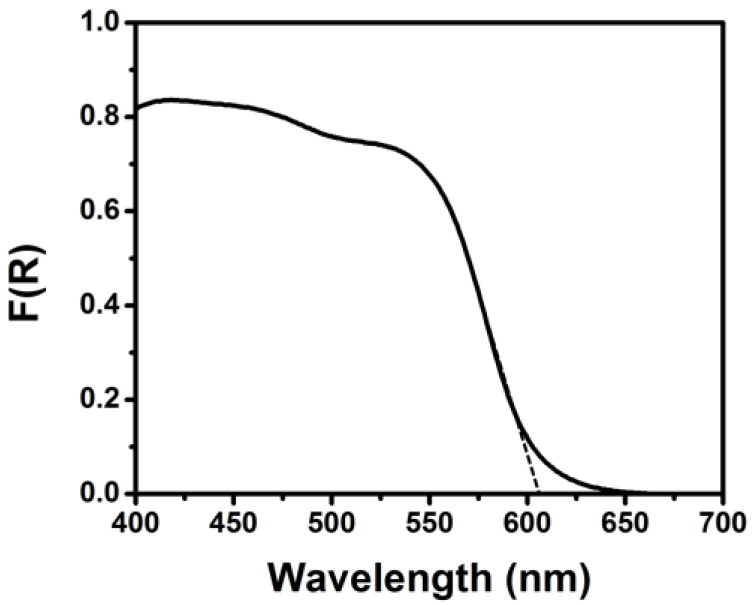
Reflection–absorption spectrum of the ZnS/CdSe/CdS/TiO_2_/fluorine doped tin oxide (FTO) photoanode electrode.

The operation of the PFC is schematically illustrated in Figure 1. Light enters through the transparent FTO electrode and it is absorbed by all the subsequent TiO_2_, CdS, CdSe and ZnS layers. The absorption spectrum sums up to the one presented in Figure 2, with an absorption onset at 610 nm (2.03 eV). This corresponds to substantial photon harvesting and is expected to provide sufficient current for the present application. Indeed, the corresponding maximum current density expected for this spectral range is 13 mA cm^2−^, according to published charts (cf. [2,18]). Photons absorbed by the photoanode generate electron-hole pairs. Electrons are injected into the lowest-lying conduction band of titania while holes are injected into the highest-lying valence band of CdSe (cf. energy levels in References [19,20]). Holes are consumed by oxidizing the electrolyte which, as already said, is composed of a S^2−^/SO_3_^2−^ combination. The following reaction (Equation (4)) describes a standard oxidation scheme:

S^2−^ + SO_3_^2−^ + 2h^+^ → S_2_O_3_^2−^ (4)

However, other equivalent oxidation reactions [20] are also possible. These reactions have redox potentials that are smaller than 0.6 V vs. SHE, i.e., smaller than the potential of the valence band of CdSe (about 1.5 V vs. SHE); therefore, oxidation of these species is always possible by the present semiconductors. The S^2−^/SO_3_^2−^ mixture is a model waste of wastes produced by oil refineries. Therefore, the photogenerated holes are consumed at the same time consuming an industrial waste. The photogenerated electrons which escape recombination are led to the air-diffusion cathode electrode where they participate in oxygen reduction. We have previously found that oxygen reduction by the present PFC is accompanied by substantial hydrogen peroxide production [17]. Therefore, we believe that oxygen reduction in the present case may to a large extent be carried out by the following reaction:

O_2_ + 2H^+^ +2e^−^→H_2_O_2_   E_0_ = +0.68 V vs. SHE (5)

This assumption is supported by the fact that the cathode electrode carries only the hydrophobic carbon layer without any powerful electrocatalyst, which would support the standard oxygen reduction reaction:

O_2_ + 4H^+^ +4e^−^ → 2H_2_O   E_0_ = +1.23 vs. SHE (6)

The photogenerated electrons are injected, as already said, into the host conduction band of titania, which lies at an energy level approximately equal to E^0^ = −1.0 V, in an alkaline environment, as it is the case with the S^2−^/SO_3_^2−^ electrolyte in the anode compartment. The pH of the Na_2_SO_4_ electrolyte in the cathode compartment is around 6; therefore, the corresponding potential of Equation (5) in such an electrolyte is around E^0^= +0.33 V. The difference between these potentials is approximately equal to 1.33 V; therefore, there is sufficient bias to drive electrons from the photoanode to the counter electrode and carry out oxygen reduction. Consequently, the cell is expected to run by itself as a solar cell without any external bias. This is verified by experiment, as shown in the next paragraph.

**Figure 3:**
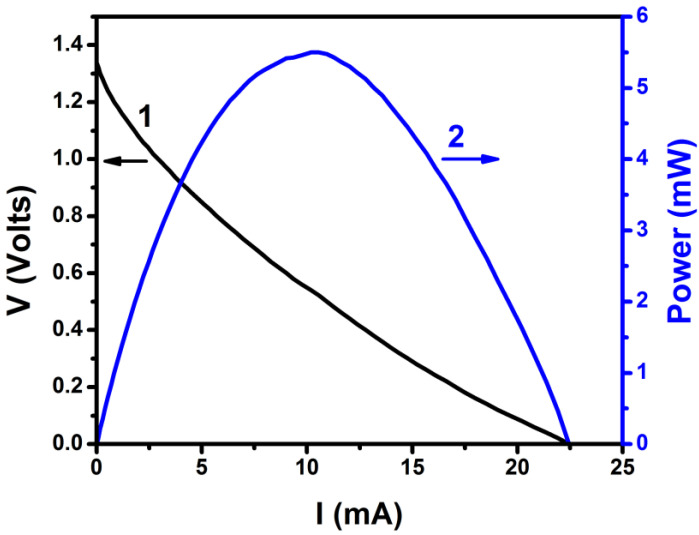
Current-voltage and power curve for the PFC of the present work. The cell consisted of 2 compartments and carried a ZnS/CdSe/CdS/TiO_2_/FTO photoanode and a carbon black (CB)/carbon cloth (CC) cathode. The aqueous electrolytes contained 0.25 M Na_2_S and 0.125 M Na_2_SO_3_ in the anode compartment and 0.5 M Na_2_SO_4_ in the cathode compartment. The active area of both electrodes was 3 cm^2^.

The current–voltage curve for the PFC used in the present work is given in Figure 3. The cell produced an open circuit voltage of more than 1.3 V and a short circuit current of more than 20 mA, under illumination. The value of the open circuit voltage fits the electrochemical potential of Reaction (5), as discussed in the previous paragraph, and thus verifies that the present PFC mainly operates by a two-electron oxygen reduction. The electric characteristics of the PFC then meet the prerequisites for charging a VRB.

**Figure 4:**
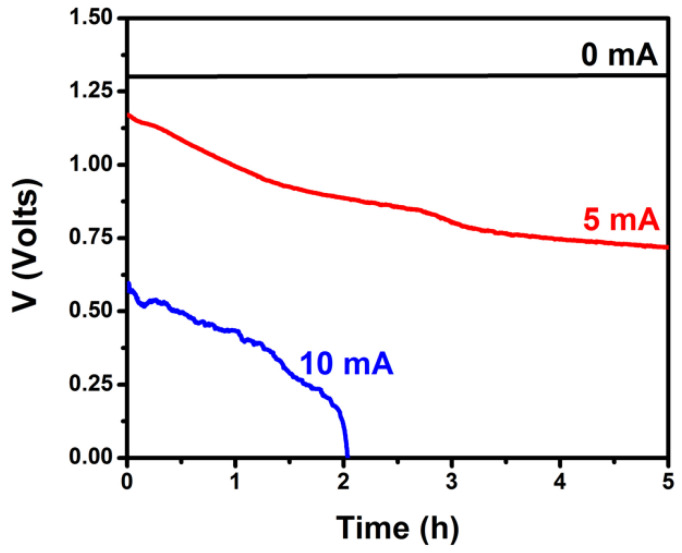
Variation of the voltage produced by the PFC under galvanostatic conditions by continuous illumination by a constant light intensity. The cell consisted of 2 compartments and carried a ZnS/CdSe/CdS/TiO_2_/FTO photoanode and a CB/CC cathode. The aqueous electrolytes contained 0.25 M Na_2_S and 0.125 M Na_2_SO_3_ in the anode compartment and 0.5 M Na_2_SO_4_ in the cathode compartment. The active area of both electrodes was 3 cm^2^.

It is furthermore necessary to obtain stability data for the PFC. The characteristics of the cell described by Figure 3 must be preserved for a period of time long enough to satisfy the demanded supply of charge. To this end, the PFC was subjected to galvanostatic measurements of the supplied voltage for several hours and for a few chosen current values. The obtained data are plotted in Figure 4. The supplied voltage was stable when the current was zero or relatively small (<1 mA). Voltage dropped by about 30% in a period of 5 h when the current was 5 mA and the cell collapsed in about two hours when the requested current was 10 mA. Voltage drop is related mainly with the consumption of the fuel according to Equation (4) or other related oxidation schemes. This was verified by refilling the cell with fresh electrolyte. Indeed, the original cell parameters were recuperated in that case. As it will be seen below, the current flow to charge the VRB was 0.2 mA, i.e., it was below 1 mA and belongs to the first case; therefore, the present PFC suffices for the intended application.

**Figure 5:**
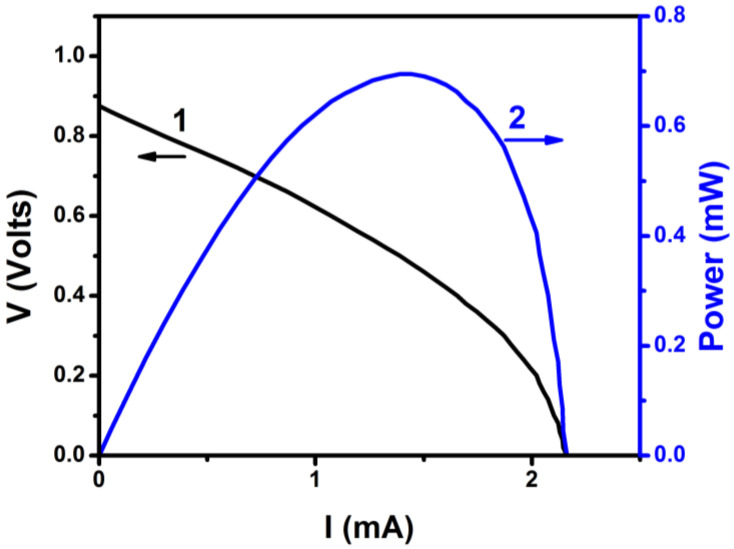
Current-voltage and power curve for a VRB directly charged by connection to the PFC.

The final step was then to connect the VRB to the PFC. The VRB was filled with the same fresh electrolyte (1 M VOSO_4_ and 2 M H_2_SO_4_) in both compartments. One electrode was connected to the photoanode and the second to the air (cathode) electrode (cf. Figure 1). The flowing current was monitored momentarily by using an amperometer. The maximum recorded current was 0.2 mA. After two hours, the color of the electrode solutions changed in both battery compartments and the VRB was charged [21]. Verification of battery charging was made by monitoring its own current-voltage characteristics, which are shown in Figure 5. The open circuit voltage of the battery was smaller than the theoretical value of 1.259 V (cf. Equations (1) and (2)) but this is expected due to inevitable losses. In any case, battery open circuit voltage was substantially smaller than the corresponding value for PFC; therefore, all conditions were satisfied and unmediated charging has been achieved. It must be noted at this point that flow of 0.2 mA for two hours is not sufficient to fully convert (i.e., fully oxidize/reduce) 40 mL of 1 M VOSO_4_ but the essential is that charging of the battery with a PFC is feasible.

## 4. Conclusions

The PFC presented in this work possessed satisfactory electric characteristics and it was capable of unmediated charging of a vanadium redox battery. All components are characterized by simplicity and easiness of construction. The photocatalytic PFC–VRB system represents one fine example of effective storage of solar energy in a high density, simple and reversible chemical energy storage system.
